# Overweight in Swedish show dogs–prevalence and association with performance in competition

**DOI:** 10.1186/s13028-021-00582-2

**Published:** 2021-04-26

**Authors:** Sanna Lindåse, Tilda Feltenmark, Malin Krantz, Josefin Söder

**Affiliations:** Department of Clinical Sciences, Faculty of Veterinary Medicine and Animal Science, Swedish University of Agricultural Sciences, Box 7054, 75007 Uppsala, Sweden

**Keywords:** Body condition score, BCS, BCS assessment, Canine, Dog show competition, Obesity, Overweight prevalence, Show dogs

## Abstract

**Background:**

The prevalence of overweight and obesity is increasing in companion dogs, but little is known of these conditions in show dogs. This study assessed body condition score (BCS) of show dogs of six selected popular breeds at a major Swedish dog show event and examined the association between BCS and performance in competition.

**Results:**

At one of Sweden’s largest dog shows, BCS of 120 dogs of six different breeds was assessed by trained animal healthcare personnel, using a 9-point BCS scale with conditional cut-off for overweight set to BCS ≥ 6. Prevalence of overweight in the cohort was 32% but all overweight dogs except one displayed only slight overweight (BCS 6) and no dog was assessed as obese (BCS 8–9). Prevalence of overweight differed significantly between breeds (P < 0.0001) with Labrador retrievers, Golden retrievers and French bulldogs showing the highest mean BCS (5.6–5.7) and highest prevalence of overweight (50–67%). Lean and overweight dogs received awards and higher show awards (certificates) to the same extent, and no significant association between slight overweight and performance in competition was found.

**Conclusions:**

Prevalence of overweight in Swedish show dogs was relatively high and in the same range as in the Swedish dog population as a whole. Dog owners, breeders and judges should be made aware of canine obesity problems and trained in BCS assessment, to better prevent canine overweight and associated health risks. This is particularly important for retriever and brachycephalic breeds, which showed high prevalence of slight overweight and have breed-specific health problems exacerbated by overweight. Owners and breeders of traditionally sturdy dog breeds should be informed that overweight dogs do not outperform lean dogs in competition.

**Supplementary Information:**

The online version contains supplementary material available at 10.1186/s13028-021-00582-2.

## Background

Obesity is a severe problem in today’s society for both dogs and humans [1]. A survey of 11 European countries found that 30–70% of companion dogs and 20–50% of dog owners were overweight [2], while studies in Scandinavia, Australia and the USA report an overweight prevalence of 20–40% in pet dogs [3–5]. There is also a high prevalence of overweight among pet dogs in Sweden, 30-50% according to estimates by dog owners [2] and animal healthcare personnel [6] in 2017–2018.

Excess canine adiposity leads to a shorter life span [7–9] and is linked to a number of different comorbidities, such as respiratory dysfunction [10–12], orthopaedic disease [8, 13] and metabolic dysfunction [14–16]. In addition, breed-specific health problems can be exacerbated when dogs are overweight or obese. For example, overweight or obesity in brachycephalic and miniature breeds increases the risk of brachycephalic obstructive airway syndrome [12, 17] and intervertebral disc disease [18–21]. Working breeds, such as Labrador retrievers and Golden retrievers, are at increased risk of hip dysplasia [22, 23] and osteoarthritis [24], and the risk and severity of these joint diseases have been shown to increase with increased adiposity [8, 24–26].

Dog shows are popular events where dog owners and breeders have the performance and presentation of their dogs evaluated by certified judges. Dogs are assessed based on the breed standard defined by the Swedish Kennel Club (SKK), which includes historical background, overall impression, health, phenotype and temperament of each dog breed (https://www.skk.se). The best-performing dogs are awarded and can also receive higher show awards (certificates) in the dog shows, and may thus set the standard for other dogs of the same breed, or dog show merits may be used in selection of parent animals. A few studies have assessed body condition score (BCS) in show dogs and have found that 20–60% of participating dogs may be overweight [27–29]. To our knowledge, no previous investigation has been performed on BCS in Swedish show dogs, so the prevalence of overweight or obese dogs among Swedish show dog populations is unknown. The extent to which overweight dogs are awarded prizes in dog show competitions is also unknown. The aims of the present study were to assess BCS of show dogs of six selected popular breeds at a major Swedish dog show event and to examine the association between BCS and performance in competition.

## Methods

### General study design

The clinical part of the study was performed by two students in their last semester in the Veterinary Nursing degree programme at the Swedish University of Agricultural Sciences. It was conducted at one of Sweden’s largest dog shows, where 120 dogs of six different breeds were body condition scored. This was a convenience sample and was not based on power analyses, although a post-hoc power calculation was performed. The BCS assessment was performed using the validated 9-point BCS scale [30], where BCS 1–3 is considered underweight, BCS 4–5 is considered lean, BCS 6–7 is considered overweight and BCS 8–9 is considered obese. The conditional cut-off for overweight (BCS ≥ 6) suggested by the scoring scale was applied to categorise dogs as lean or overweight. All assessments were made by one of the two students who, prior to the study, received training in BCS assessment and specific BCS calibration provided by an experienced BCS assessor (JS). Written consent on participation was obtained from the owners of all dogs. The consent included permission to assess BCS, permission to access the dog’s registration number and permission to record personal data for scientific purposes. The study was conducted under animal ethics research permit (No. 5.818-15533/2018) issued by the Ethics Committee for Animal Experiments, Uppsala, Sweden.

### Breeds evaluated and processing of dog show results

The choice of breeds to include in the assessment was based on Sweden’s 20 most popular dog breeds according to statistics on newly registered dogs in 2020 published by SKK [31]. Some of the breeds on the list were under-represented in the selected dog show and were therefore not included in the study. Only three pugs were assessed in the study (all BCS 6) and these were excluded from the statistical analyses as they were too few to represent the breed. The breeds included in the study were; chihuahua, dachshund, French bulldog, Golden retriever, Labrador retriever and whippet. The whippet is not one of Sweden's 20 most popular dog breeds, but was added to give a wider variety of dog types in the sample. Individual competition results from the dog show were obtained from the SKK database and the results were scored as follows: Did not receive a judgement (0), good (1), very good (2) and excellent (3). The dogs were also divided into two groups depending on their performance, where dogs rated excellent were classified as ‘award’ and those less than excellent as ‘no award’. Only individual dogs scored excellent were allowed to continue in their competition and the cut-off was therefore chosen accordingly. Information on whether individuals rated excellent received an additional award (‘certificate’ or ‘no certificate’) indicating even higher quality performance was also recorded.

### Statistical analyses

First, the Mann–Whitney U test was used to explore whether male and female dogs differed in BCS or in age. Second, linear regression analysis was used to explore whether increasing age was positively associated with increasing BCS for all dogs, regardless of breed. Thereafter, linear regression analysis was used to investigate whether increasing BCS was positively associated with increasing rating (0–3) in competition for all dogs, regardless of breed. A Chi-square test was used to analyse whether the prevalence of overweight dogs (BCS ≥ 6) within each breed differed between the six breeds studied. Lastly, Fisher’s exact test was used to analyse whether performance in competition (‘award’ versus ‘no award’) depended on BCS (‘lean’ versus ‘overweight’) within each breed and Fisher’s exact test was used to analyse whether receiving a certificate (‘certificate’ versus ‘no certificate’) depended on BCS (‘lean’ versus ‘overweight’) for all dogs, regardless of breed. The software GraphPad Prism 5.0 (San Diego, CA, USA) was used for data analyses and statistical significance was set to P < 0.05 for all analyses.

## Results

Of the 120 dogs that were body condition scored in the study, 49 were males and 71 were females. A total of 38 dogs were assessed as having BCS ≥ 6, which corresponded to an overweight prevalence of 32% in the study population. However, all overweight dogs except one showed only slight overweight (BCS 6). There were no differences in mean BCS ± SD (5.2 ± 0.6 and 5.3 ± 0.6, P = 0.18) or in mean age ± SD (2.5 ± 2.2 and 2.7 ± 2.8, P = 0.76) between male and female dogs, and no association was found between age and BCS in linear regression analysis (P = 0.17) with all dogs included. Descriptive statistics on sex and age of each selected breed are shown in Table 1.

**Table 1:** Descriptive statistics on sex, age and prevalence of overweight in the six selected breeds and in all 120 dogs included in the study

Breed	Sex	Years	Prevalence of overweight (%)***
	(M/F)	(Mean ± SD)	BCS ≥ 6
Chihuahua	9/13	1.4 ± 0.9	32
Dachshund	8/14	2.8 ± 3.3	5
French bulldog	9/3	1.2 ± 0.6	50
Golden retriever	7/16	4.1 ± 3.0	57
Labrador retriever	4/11	2.4 ± 1.4	67
Whippet	12/14	2.7 ± 2.6	4

*BCS* Body condition score, *F* Female, *M* Male, *SD* Standard deviation

^***^The prevalence of overweight dogs (BCS ≥ 6) within each breed differed between breeds (Chi-square test, P < 0.0001)

### Comparisons of BCS among breeds

**Fig. 1:**
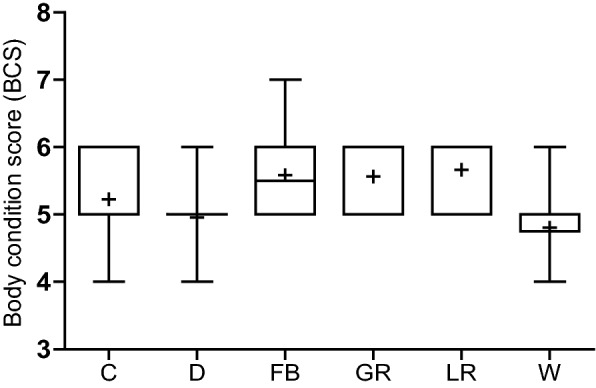
Boxplot distribution of body condition score (BCS) within each breed. Dogs were assessed according to the 9-point BCS scale, where dogs with BCS 4–5 were considered lean and dogs with BCS ≥ 6 were considered overweight (total number of dogs n = 120). Values are given as mean (cross), 25% and 75% percentile (box), and min/max (whiskers). Breed shown on x-axis; chihuahua (C), dachshund (D), French bulldog (FB), Golden retriever (GR), Labrador retriever (LR) and whippet (W)

**Fig. 2:**
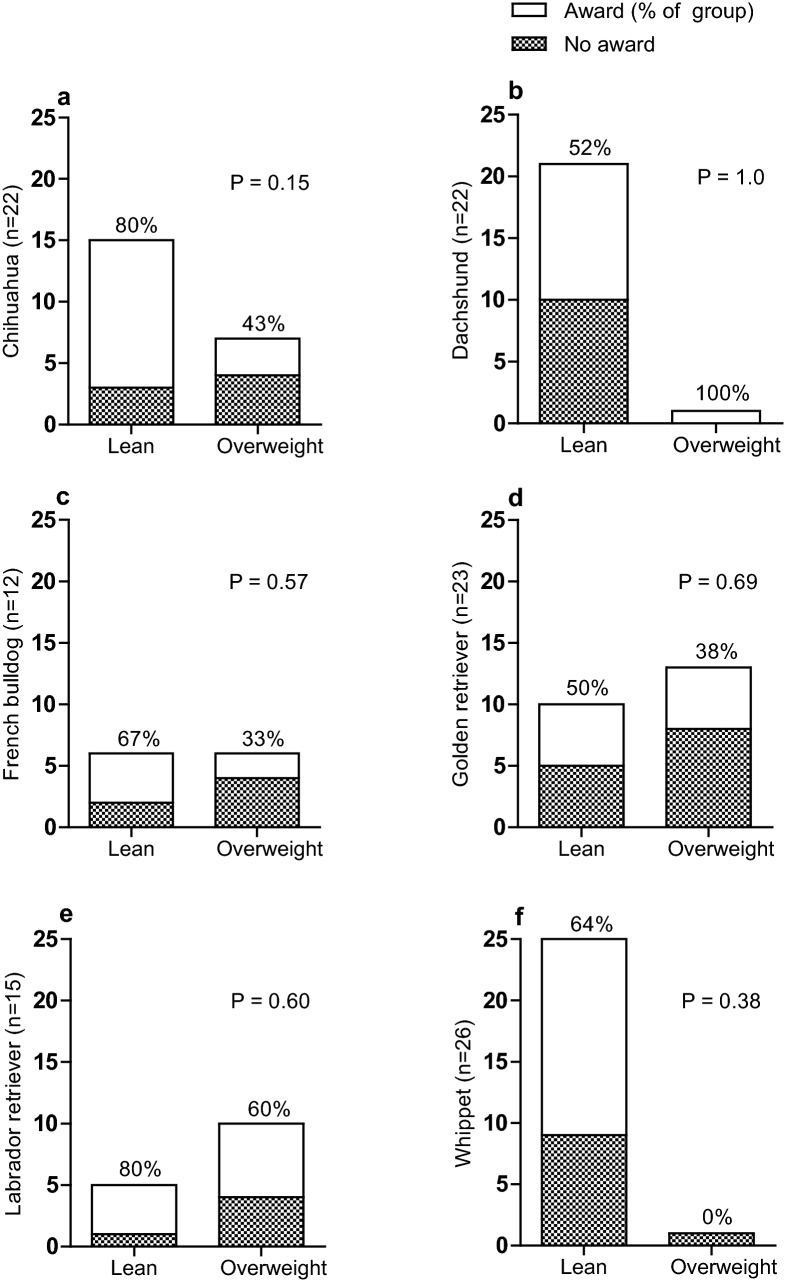
Body condition score (BCS) and performance in competition of each selected breed. Dogs were assessed according to the 9-point BCS scale, where dogs with BCS 4–5 were grouped as lean and dogs with BCS ≥ 6 were grouped as overweight. (**a**–**f**) Bar chart for each breed. Performance in competition (‘award’ versus ‘no award’) did not depend on BCS (‘lean’ versus ‘overweight’) within each breed (Fisher’s exact test, P-values in diagrams)

Body condition in the 120 dogs assessed ranged from BCS 4–7 and no dog was scored as underweight (BCS 1–3) or obese (BCS 8–9) (Fig. 1). The prevalence of overweight dogs (BCS ≥ 6) within each breed differed significantly between breeds (P < 0.0001) (Table 1). The breed with the highest mean BCS was Labrador retriever, followed by Golden retriever and French bulldogs. In those three breeds, no dog was assessed as being in the lower range of lean (BCS 4) and overweight dogs were equal to or outnumbered lean dogs. The breed with the lowest mean BCS was the whippet, followed by the dachshund, and in those breeds, there were almost no overweight dogs (Figs. 1, 2).

### Association of BCS with performance in competition

**Fig. 3:**
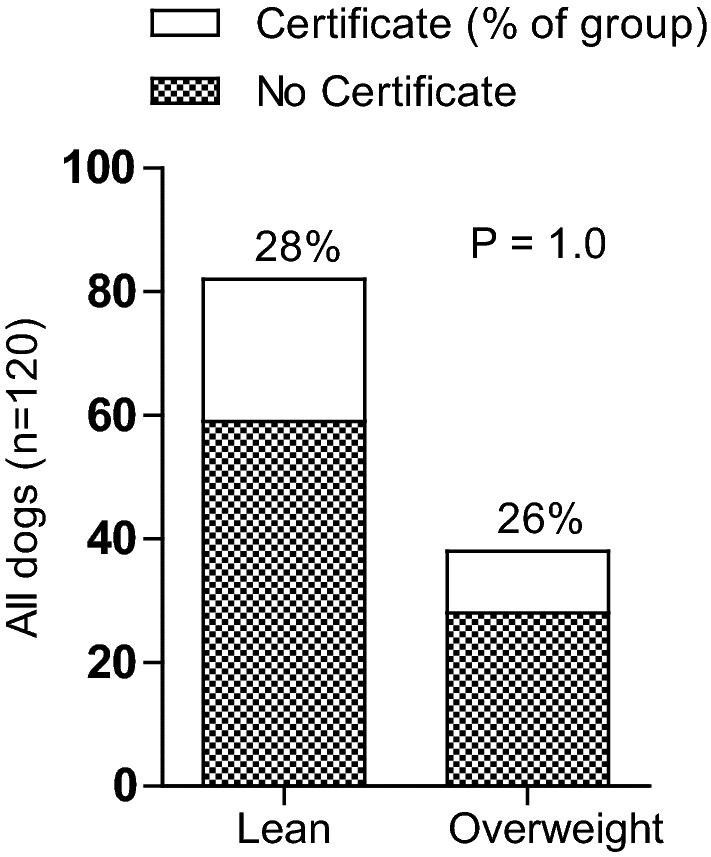
Body condition score (BCS) and performance in competition of all dogs. Dogs were assessed according to the 9-point BCS scale, where dogs with BCS 4–5 were grouped as lean and dogs with BCS ≥ 6 were grouped as overweight. Performance in higher levels of competition (‘certificate’ versus ‘no certificate’) did not depend on BCS (‘lean’ versus ‘overweight’) for all dogs included (Fisher’s exact test, P-value in diagram)

No association was found between BCS and performance in competition (score 0–3) for any of the dogs, regardless of breed, in linear regression analysis (P = 0.54). Performance in competition (‘award’ versus ‘no award’) did not depend on BCS (‘lean’ versus ‘overweight’) within each breed (Fisher's exact test, P > 0.05) (Fig. 2). Moreover, higher performance in competition (‘certificate’ versus ‘no certificate’) did not depend on BCS (‘lean’ versus ‘overweight’) for any of the dogs included (Fisher’s exact test, P > 0.05) (Fig. 3).

## Discussion

Few previous studies have reported BCS assessments of show dogs. This is the first study to perform BCS assessments on Swedish show dogs and is also the first to examine the association of BCS with their performance in competition. The results showed relatively high prevalence of overweight (32%) in the study population of show dogs. However, all overweight dogs except one was only slightly overweight (BCS 6) and no dog was assessed as being obese (BCS 8–9). The prevalence of overweight differed between breeds, with the retrievers and the brachycephalic breeds showing the highest prevalence and the highest mean BCS. Whippets and dachshunds, on the other hand, showed almost no overweight individuals. In this study, lean and overweight dogs received awards and certificates to the same extent, and no association between BCS and performance in competition was found.

A significant difference in the prevalence of overweight between breeds was detected. Labrador retriever and Golden retriever were the breeds with the highest prevalence of overweight, followed by French bulldog. In those breeds, half of the population or more was overweight and in the Labrador retriever breed 67% were overweight. In the study population of Swedish show dogs, prevalence of overweight was as common as in companion dogs overall in Sweden, based on a survey of Swedish dog owners in 2018 [2]. The results from the present study cannot be directly applied to the entire Swedish show dog population, as they are limited to a restricted number of individuals in a few specific dog breeds, but they provide an indication of the severity of the problem.

Examination of the association between BCS and performance in competition showed that lean and overweight dogs were given awards to the same extent and that the judges did not favour either lean or overweight individuals. However, there were numerical differences between the breeds, with chihuahuas and French bulldogs showing a tendency to receive awards more frequently if they were lean and a numerically higher number of overweight Labrador retrievers were awarded. Those tendencies within breeds were not significantly different in the statistical analyses, either because there were no true differences or because of power problems as the groups of lean and overweight dogs were in general on the border of power or below in post-hoc power calculations. From an animal healthcare perspective, it would be preferable if no overweight dogs received an award from dog show judges, as this could encourage owners to keep their dogs in excess body condition, despite known health risks, if they perceive that judges prefer ‘sturdy’ dogs. Informing breeders and dog owners that judges do not give more awards to sturdy dogs could be a good way to encourage dog keepers to keep their show dogs in lean body condition.

A limited number of previous studies have assessed BCS in show dogs. One study performed by a veterinarian in the Netherlands in 2012 found that 18% of participating show dogs were overweight [27]. Overweight in companion dogs is generally perceived to have increased in recent years, and obesity is now considered a disease in pets [32]. It is likely that the overweight prevalence in show dogs has increased simultaneously, as indicated by our results for Sweden. However, the mean BCS of individual breeds included in the study from the Netherlands and in the present study was similar and both studies showed that the prevalence of overweight varied between different breeds. A study in 2015 assessing BCS of show dogs in the United Kingdom (UK) using images [28] reported that 26% of all dogs assessed were overweight, but there was a slight difference in breeds found to be overweight compared with the present study. That study found that Labrador retrievers were strongly affected, as in Sweden, but Golden retrievers displayed a lower prevalence of overweight compared with in the present study. The UK study found overweight dachshunds [28], which the present study could not confirm. These discrepancies may be due to genetic differences between breeds in Sweden and the UK, or to differences in the assessment method used. The Golden retriever is a relatively long-coated breed and this might have resulted in under-estimation in BCS assessments performed on images, rather than conventional palpation, which was raised as a limitation of using the image method in the study [28]. A later study in the UK, in 2018, found a dangerous trend in pet obesity where 56% of show dogs assessed were overweight and 7% were obese [29]. That study found overweight and obesity even in juvenile dogs and increasing prevalence of overweight with increasing age in the study cohort, findings that the current study could not confirm.

Discrepancies in overweight prevalence in show dogs in the different cohorts described may also be due to inclusion of separate breeds. The present study included at least one breed that generally tends to be lean, the whippet [27], which might have had an impact on the total overweight prevalence in the Swedish cohort. The whippet is not among the 20 most popular breeds in Sweden, but was included to give wider dog type diversity. On excluding the results for this breed, the overweight prevalence in the cohort in the present study was much higher (40%). Differences in overweight prevalence between studies of show dogs in different countries may also derive from national differences in the judges’ view of ‘ideal’ body condition of a show dog, which may influence the BCS in which the dogs are kept. The differences may also depend on discrepancies between BCS assessors, although the BCS system generally shows good inter-assessor agreement [30, 33]. Before more general conclusions can be drawn on the prevalence of overweight in show dogs in Sweden, there is a need for further studies of larger cohorts including breeds other than those assessed in the present study.

This study revealed quite widespread excess adiposity in Swedish show dogs, but it should be emphasised that almost all of the dogs categorised as overweight were only slightly overweight (BCS 6) and that no dog was obese (BCS 8–9). The BCS assessment method is a clinical method based on palpation and visual inspection of different characteristics known to be correlated with total body fat percentage in dogs [30]. Canine overweight can be viewed as a binary variable (a dog is either lean or overweight), but in reality canine overweight is a continuous variable ranging from slight overweight to severe obesity, with the total body fat percentage increasing with each point on the BCS scale [30, 34]. The 9-point BCS scale has been validated by dual-energy X-ray absorptiometry (DEXA) and is commonly used in research, but a 5-point BCS scale is also used [34]. The ability to identify dogs with slight overweight depends on the scale and the skill of the assessor. Slightly overweight dogs risk being falsely classified as lean when the 5-point BCS scale is used, as that scale does not identify dogs with slight overweight unless the assessor uses half points on the scale [2, 35]. Therefore, the 9-point BCS scale should be used in training assessors, to increase identification and awareness of slight overweight in dogs.

Being overweight affects quality of life, life span and the occurrence of chronic diseases in dogs [8, 36], but few studies have so far considered the adverse effects of slight overweight. It has been demonstrated that overweight may reduce the life span of a dog by two years or more [7–9]. However, that finding was made in longitudinal studies concentrating on prominently overweight or obese dogs excluding slightly overweight dogs as a group. It is therefore unknown whether the latter group of dogs risks a shorter life or not. In one of the longitudinal studies cited [8], the overweight group had mean BCS of only 6.7 and the life span of those dogs was in general two years shorter than that of their lean paired-matched partners. Based on those results, it is possible that dogs with slight overweight throughout life risk a shorter life span, so longitudinal studies of this particular group of dogs are warranted. In addition to more general health problems linked to overweight, some breed-specific health-problems are exacerbated by increased BCS. A notable finding in the present study was that breeds characterised by such specific health problems had the highest prevalence of overweight in the cohort. Brachycephalic breeds such as pugs and French bulldogs have respiratory problems that are intensified by prominent overweight (BCS ≥ 7)[12, 17], but it is not yet known whether slight overweight also has a negative impact on breathing characteristics in brachycephalic breeds. Only three pugs were assessed in this study, but all were slightly overweight (BCS 6), indicating a need for further investigations on BCS of show pugs. In Golden and Labrador retrievers, orthopaedic disease is common and many of the joint diseases suffered by these breeds are exacerbated by overweight [8, 24–26]. In fact, body weight at or above the breed mean in Golden and Labrador retrievers has been shown to be a risk factor for osteoarthritis [24]. In a longitudinal study, overweight Labrador retrievers (mean BCS 6.7) started treatment for osteoarthritis two years earlier than their lean paired-matched partners [8]. In the cohort of show dogs assessed in the present study, the retriever breeds showed high prevalence of slight overweight and, although the exact BCS cut-off for increased risk of orthopaedic diseases in dogs has not been firmly established, slight overweight might be a risk factor. It is important to have lean body condition in all dog breeds, but particularly in breeds whose health is known to be more adversely affected by overweight.

Various studies have shown that appropriate training is needed to accurately assess BCS in dogs. For example, dog owners tend to under-estimate the body condition of their dogs [35, 37, 38], even if they have been instructed to use a BCS scale [37]. Identifying a slightly overweight dog (BCS 6) can be challenging even for an experienced assessor, as this individual has not yet developed marked fat deposits. Such a dog is described by the 9-point BCS scale as “*Ribs palpable with slight excess fat covering. Waist is discernible viewed from above but is not prominent. Abdominal tuck apparent*” [30]. Identification of dogs with slight overweight is of high importance, as often only small interventions are needed to get these dogs back into lean condition. If slight overweight remains undetected, it can easily tip over into prominent adiposity or obesity, which can be much more challenging to treat [39, 40]. Different assessors may differ slightly in their BCS rating for the same dog [30], but clinical BCS assessment is generally a quick and valuable tool in research and in clinical settings [34]. Inter-observer variability in scoring of the present cohort was minimised by having the two assessors trained similarly.

The dogs included in the cohort were a convenience sample reflecting the number of dogs that the two trained veterinary nurses could assess during the dog show. Assessments at an additional dog show were originally planned, but the event had to be cancelled due to the Coronavirus pandemic. Post-hoc power analysis based on descriptive data for the dog breeds with the highest and lowest mean BCS ± SD showed that a sample size of at least seven dogs in each group gave acceptable power (power 0.8, α 0.05). Based on this calculation, the three pugs were excluded from the statistical analyses and diagrams. As unequal group sizes will not affect the incidence of type I errors, the observed difference in prevalence of overweight between the six breeds should be interpreted as a valid result, particularly as enough dogs were included within each breed according to the power analysis. However, type II errors are affected by unequal group sizes, and greater sample size could possibly have revealed differences otherwise not detected. There were no significant differences in proportions of lean and overweight dogs within breeds receiving awards in competition, but further testing using greater sample sizes is required to confirm this, especially for dachshunds and whippets, where almost no dog was overweight.

High-performing show dogs may set the physical standard for other dogs of the same breed. In the Swedish breed standards (http://www.skk.se) of the brachycephalic and retriever breeds included in this study, physical attributes that might be created by excess adiposity are described [41]. For example, according to the SKK breed standard retrievers should have “a wide lumbar area”, which could be interpreted as absence of a waist, and French bulldogs should have “a heavy bone structure”. All brachycephalic and retriever breeds included in the study are described in the standards as “robust”, “compact” and with “pronounced muscle mass”. With those descriptions, it is important that judges are not misled by overweight and correctly distinguish muscle mass from adipose tissue, to adequately reward lean individuals in good shape. This distinction is not trivial and requires visual inspection and palpation for BCS assessment and preferably also for muscle mass assessment of each competing dog. The Swedish breed standard for the Labrador retriever breed explicitly states that certain physical attributes should not be achieved through excess adiposity, but 67% of the Labrador retrievers in the dog show studied were slightly overweight (although none was prominently overweight). It is difficult to determine whether descriptions in the breed standards are associated with higher prevalence of overweight in retrievers and French bulldogs. Canine obesity has been demonstrated to be a multi-factor problem [42], involving dog-related factors such as breed genetics and dog owner-related factors such as feeding and exercise routines [2, 38, 43, 44]. Overall, however, the Labrador retriever, Golden retriever and pug breeds have been shown to display a higher prevalence of overweight than other breeds in studies from different continents [5, 38, 43, 44]. In addition, the Labrador retriever, traditionally described as greedy, has a mutation of the pro-opiomelanocortin (POMC) gene present in approximately 22–45% of the breed population [45]. This genetic mutation modulates substances mediating in the appetite centre, making the dog more food-motivated and increasing the risk of overweight. The Labrador retriever could therefore be more difficult to keep lean than other breeds, irrespective of the description in the breed standard and partly irrespective of owner-management differences.

Companion dogs are often regarded as family members and often share a lifestyle with their owners [1]. It has been shown that the owner’s education, family income, parenting style and attitude towards healthy food and physical activity may influence the BCS of a dog [2, 46]. Awareness of obesity is another important factor to address, as dog owners who do not consider obesity a disease are more likely to have an overweight dog [2]. The high prevalence of overweight detected in Swedish show dogs in this study may be because dog owners, breeders and judges try to meet the breed standards by keeping show dogs of certain breeds slightly overweight, or may reflect lack of awareness of the canine obesity problem and lack of training in BCS assessment. If BCS assessment is performed in evaluation of a show dog probably vary between judges, between dog shows and between countries. The qualification programme for Swedish dog show judges includes a course in canine anatomy, but no specific training in obesity-related health problems or how to perform BCS assessment. The general body condition of the dog must be included in the overall impression, but a BCS scale is not used for that purpose (SKK, personal communication 2021). The judges at the particular Swedish dog show studied had 9–26 years of experience and had judged a total of 40 000 dogs in Sweden. All had all judged major shows before and most had judged internationally as well as nationally. The judges, who must thereby be regarded as very experienced, gave awards to lean and overweight dogs to the same extent. It may thereby be important to explore if and how BCS is included in the overall impression and judgement of lean and overweight dogs. Judges’ ability to assess BCS in show dogs should be investigated in further research, as it is critical that even slight overweight is addressed and that judges, breeders and dog owners work together as a team to maintain canine health and well-being.

## Conclusions

Prevalence of overweight in Swedish show dogs was relatively high (32%), consisted of slight overweight and was in the same range as in the Swedish dog population as a whole. Labrador retrievers, Golden retrievers and French bulldogs showed the highest mean BCS (5.6–5.7) and highest prevalence of overweight (50–67%) but lean and overweight dogs received awards to the same extent, and no significant association between slight overweight and performance in competition was found. Dog owners, breeders and judges should be made aware of canine obesity problems and trained in BCS assessment, to better prevent canine overweight and associated health risks. This is particularly important for retriever and brachycephalic breeds, which showed high prevalence of slight overweight and have breed-specific health problems exacerbated by overweight. Owners and breeders of traditionally sturdy dog breeds should be informed that overweight dogs do not outperform lean dogs in competition. For more general conclusions on overweight prevalence in show dogs in Sweden and their performance in competition, further studies of larger cohorts including other breeds are required.

## Supplementary Information

**Additional file 1.** Raw data (breed, sex, age, BCS and performance in competition) for the 120 dogs included in the study cohort.

## Data Availability

The dataset supporting the conclusions of this article is included within the article and its additional file.
